# Comprehensive Evaluation of Hepatitis E Serology and Molecular Testing in a Large Cohort

**DOI:** 10.3390/pathogens9020137

**Published:** 2020-02-19

**Authors:** Olympia E. Anastasiou, Viktoria Thodou, Annemarie Berger, Heiner Wedemeyer, Sandra Ciesek

**Affiliations:** 1 Institute of Virology, University Hospital of Essen, University Duisburg-Essen, 45147 Essen, Germany; olympia.anastasiou@uni-due.de; 2 Department of Gastroenterology and Hepatology, University Hospital of Essen, 45147 Essen, Germany; Viktoria.Thodou@uk-essen.de (V.T.); Heiner.Wedemeyer@uk-essen.de (H.W.); 3 Institute for Medical Virology, University Hospital, Goethe University Frankfurt am Main, 60590 Frankfurt, Germany; annemarie.berger@em.uni-frankfurt.de; 4 German Center for Infection Research, DZIF, 38124 Braunschweig, Germany

**Keywords:** hepatitis E, HEV, serology, PCR, immunosuppression

## Abstract

Introduction: Reliable and cost-effective diagnostics for hepatitis E virus (HEV) infection are necessary. The aim of our study was to investigate which diagnostic test is most accurate to detect HEV infection in immunocompetent and immunosuppressed patients in a real world setting. Patients and Methods: We performed a retrospective analysis of 1165 patients tested for HEV antibodies and HEV PCR at the same time point. Clinical, laboratory and virological data were taken from patient charts. HEV IgA was measured in a subgroup of 185 patients. Results: HEV RNA was detectable in 61 patients (5.2%); most of them (n = 49, 80.3%/n = 43, 70.5%) were HEV IgM+ and IgG+; however, 12 patients (19.6%) were HEV RNA positive/HEV IgM negative and 17 patients (27.8%) were HEV RNA positive/HEV IgG negative. Ten HEV RNA positive patients (16.4%) had neither HEV IgG nor IgM antibodies. Importantly, all of them were immunosuppressed. HEV IgA testing was less sensitive than HEV IgM for HEV diagnosis. Conclusions: HEV infection can be overlooked in patients without HEV specific antibodies. Performing PCR is necessary to diagnose or exclude HEV infection in immunocompromised hosts. In immunocompetent patients, a screening based on HEV antibodies (IgG/IgM) is sufficient.

## 1. Introduction

Hepatitis E virus (HEV) infection is an important and emerging public health issue associated with considerable morbidity and mortality. In the past, its impact was thought to be limited to developing countries, where it caused epidemics due to water contamination and poor sanitation [1]. Today it is known to be endemic in most high-income countries as a zoonotic disease with pigs as the primary reservoir [2]. HEV infection is the major cause of acute viral hepatitis in many European countries, with more reported cases than acute hepatitis A or B [3]. Acute HEV infection is often asymptomatic in immunocompetent individuals; its clinical manifestation ranges from mild hepatitis to acute (on chronic) liver failure (A(C)LF) [2,4,5]. It has also been associated with extrahepatic manifestations, mostly neurological but also musculoskeletal, hematological and renal [6].

In the last years, our knowledge about the natural course of HEV infection has expanded. Previously, HEV was believed to cause exclusively acute and mostly self-limiting hepatitis, not unlike the hepatitis A virus (HAV). Today, we know that chronification of HEV infection is possible in immunocompromised hosts, causing significant morbidity and mortality [2].

HEV diagnosis depends on serological and molecular methods. After infection, shortly before the onset of symptoms, HEV RNA becomes detectable in the serum and stool. HEV IgM antibodies appear first and are followed soon by IgG antibodies. The IgM antibodies usually persist for 3–4 months in serum, while the IgG antibodies can be detected for a significantly longer time period [2]. The laboratory diagnosis according to the EASL guidelines relies on HEV RNA detection or a combination of serology and HEV RNA detection, but anti-HEV IgM positivity with rising anti-HEV IgG has also been proposed as a criterion [2]. The detection of anti-HEV IgM alone has been criticized as insufficient to diagnose acute HEV due to cross reactivity to cytomegalovirus (CMV) and Epstein Barr virus (EBV) [7]. False positive cases have also been reported in cases of parvovirus, leptospira and HAV infection [8,9]. According to the WHO, definitive diagnosis of HEV is usually based on the detection of specific IgM antibodies in blood, while HEV RNA detection is recommended in areas where hepatitis E is infrequent and in cases with chronic HEV infection [10]. However, reimbursement of quantitative HEV PCR is often a problem in the real world setting.

Reliable and cost-effective diagnostics for HEV infection are necessary for both low- and high-income countries. The aim of the present study is to evaluate the diagnostic robustness of HEV antibody tests (IgM, IgG and in a subgroup IgA) versus HEV PCR in a large German cohort and to investigate if immunosuppression has an impact on the reliability of the diagnostics.

## 2. Patients and Methods

**Figure 1:**
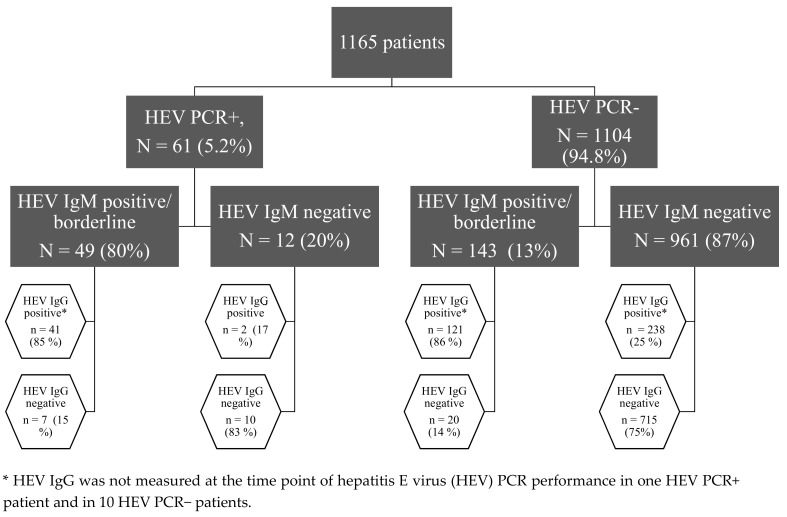
Our patient cohort stratified according to the results of HEV serology and PCR.

We performed a retrospective analysis of 1165 patients treated in the University Hospital Essen, Germany, from 2017 to 2018, most of them with hepatitis of unknown etiology. The HEV diagnostic was performed to exclude or diagnose an HEV infection. In all enrolled patients, HEV serology (HEV IgG and IgM) and HEV PCR were performed at the same time point. Patients were divided into four groups according to their HEV RNA and HEV IgM status: 1. HEV RNA+/HEV IgM+, 2. HEV RNA+/HEV IgM–, 3. HEV RNA–/HEV IgM– and 4. HEV RNA–/HEV IgM+, as shown in Figure 1. Borderline (S/CO 0.8–1.09) and positive (S/CO ≥ 1) results were grouped together; the negative results (S/CO < 0.8) formed the second group.

Routine laboratory parameters such as liver transaminases and coagulation parameters were measured in the central laboratory of the University Hospital Essen, Germany, and taken from patient charts. Total immunoglobulin G was measured in serum (Atellica CH930, Siemens, Erlangen, Germany). Virological parameters were measured in the Institute of Virology; HEV IgM and IgG, as well as HEV IgA in a subgroup of patients (*n* = 185) according to sera availability, were measured according to the manufacturer´s instructions (Anti-Hepatitis-E-Virus (HEV)-ELISA, Euroimmun AG, Germany).

HEV IgG and IgM were measured in total in 1154 and 1165 patients, respectively, as matter of clinical routine. HEV RNA levels were measured by extracting RNA using the MagNa Pure 96 Instrument (Roche, Penzberg, Germany) followed by amplification and detection with the RealStar HEV RT-PCR Kit (Altona Diagnostics, Hamburg, Germany) on the RotorGene Q (Qiagen, Hilden, Germany) according to the manufacturers’ instructions.

The patients were stratified according to the presence or absence of immunosuppression. The immunosuppressed group included patients with hematological diseases (for example chronic myeloid leukemia), patients under immunosuppressive treatment regimens for malign or benign diseases, HIV positive patients and patients after solid organ or allogeneic bone marrow transplantation. Severe hepatitis was defined as the presence of compromised coagulation with an international normalized ratio (INR) greater or equal to 1.5. Chronic HEV infection was defined as a persistence of HEV replication for three months.

This retrospective study was carried out in accordance with the Declaration of Helsinki and the guidelines of the International Conference for Harmonization for Good Clinical Practice. Informed consent was waived due to the retrospective character of the study. 

Categorical and continuous data were compared between the patient groups. Statistical significance was assessed by Chi-Square or Fisher´s exact test for categorical data. Continuous data were expressed as median (interquartile range). Unpaired t-test or Mann–Whitney U test were used to compare continuous variables. Two-tailed *P* values less than 0.05 were considered statistically significant. Statistical analysis was performed using SPSS software (v21, SPSS Inc., Chicago, IL, USA), GraphPad Prism 6.0 (GraphPad, CA, USA) and the platform VassarStats (http://vassarstats.net).

## 3. Results

### 3.1. Not All Patients with Replicative HEV Infection Had Detectable HEV Specific Antibodies

Our patient cohort is presented in Figure 1. Most patients were HEV RNA−/HEV IgM− (*n* = 961, 83%); 25% of them had serological signs of a resolved HEV infection (*n* = 238) and 13% of all patients had positive or borderline HEV IgM without detectable HEV RNA (*n* = 143). In most of these patients, we could also detect HEV IgG antibodies (*n* = 121, 83%). Our cohort included 678 (57.8%) men and 492 (42.2%) women; 74 (6.4%) of them were children and 1091 (93.6%) were adults. The average age was 52 (35–62.5) years, while 634 (54.4%) patients were immunosuppressed and 531 (45.6%) were immunocompetent. Forty one patients (3.5%) were screened for HEV (including HEV PCR) without showing biochemical or clinical signs of hepatitis in the context of potential organ, tissue or blood donation.

HEV RNA was detectable in 61 patients; in the majority of them (*n* = 49, 80%) HEV IgM antibodies were positive or borderline. However, in 20% of patients with detectable HEV RNA, HEV IgM antibodies were not detectable. A third of these patients (*n* = 4) developed HEV IgM at a later time point while the others remained negative. Furthermore, 17 patients with HEV RNA had no detectable HEV IgG antibodies (28%), and 10 patients had neither HEV IgM nor IgG antibodies (16.4%).

### 3.2. HEV Infected Immunocompetent Patients Have Higher IgG and HEV IgA Titers and Are at Higher Risk for an Acute Liver Failure Compared to Immunosuppressed Patients

It has been shown that the course of HEV infection might differ between immunocompetent and immunosuppressed patients. Therefore, our HEV RNA positive cohort was grouped into individuals according to presence or absence of immunosuppression. Thirteen immunocompetent patients were compared to 48 immunocompromised individuals, as shown in Table 1. Overall, immunocompetent patients were older (56.5 (16) vs. 51 (32) in years, *p* = 0.044), had an increased risk for liver failure (*n* = 6, 46% vs. *n* = 1, 2%, *p* < 0.001) and overall mortality (*n* = 4, 31% vs. *n* = 2, 4%, *p* = 0.016), had higher serum IgG concentration (13.1 (6.9) vs. 8.9 (5.3) g/L, *p* = 0.011) and higher HEV IgA S/CO (4.63 (2.75) vs. 1.33 (3.73), *p* = 0.012) than immunosuppressed patients in our cohort. Immunocompetent patients tended to have higher HEV IgM titers, but the difference did not reach statistical significance. In addition, alanine aminotransferase (ALT) levels were significant higher in the immunocompetent group, indicating that the immune system might play an important role in the pathogenesis of HEV infections.

### 3.3. HEV PCR Positive Patients with Negative HEV IgM Had Lower Serum IgG and Lower HEV IgG Levels

As stated above, some patients with detectable HEV RNA did not have HEV specific antibodies, which complicated diagnosis. To find out if there were specific clinical or virological characteristics explaining why some patients had HEV specific IgM antibodies while others did not, our HEV RNA positive patient cohort was divided into two groups according to their HEV IgM status. Clinical, laboratory and virological data of the subgroups are presented in Table 2. The two groups did not differ significantly in terms of age, clinical background, ALT, HEV viral load or outcome. Surprisingly, there was also no significant difference between the immunosuppressed group and the immunocompetent group. However, all patients without detectable HEV IgM and IgG (*n* = 10) were under immunosuppression (Supplementary Materials Table S1). The only significant differences were observed in the total serum IgG, HEV IgG and HEV IgA levels, which were higher in the group with detectable HEV IgM (12.1 (9.2–13.7) vs. 7.1 (6.6–8.9) g/L, *p* = 0.004, 5.32 (2.87–6.35) vs. 0.26 (0.19–0.53), *p* < 0.001 and 3.59 (1.96–4.62) vs. 0.16 (0.11–0.32), *p* < 0.001, respectively). Detailed clinical data of HEV IgM and HEV IgG negative HEV PCR positive patients are presented in Supplementary Materials Table S1.

### 3.4. HEV RNA Negative/HEV IgM Positive Patients Frequently Had No History of HEV Infection

While some patients in our cohort clearly had an HEV infection—proven by HEV RNA detection in the blood—13% of our patient cohort had HEV IgM specific antibodies without detection of HEV RNA (Figure 1). These patients were slightly older than those with undetectable HEV IgM (52 vs. 47 years old, *p* = 0.008) and were more often immunosuppressed (61.5 vs. 51.8%, *p* = 0.031, OR = 1.49 (1.04–2.13)). Furthermore, HEV IgG and IgA were more often detectable (Supplementary Materials Table S2).

As a next step, we focused on the group of HEV RNA negative HEV IgM positive patients and looked for documented history of HEV infection in patient charts. Indeed, in 20 out of 143 cases (14%), previous acute HEV infection had been documented. Interestingly, in some of these patients, the timepoint of last HEV RNA detection and first negative HEV RNA PCR was several years in the past, reaching to 4 and 6 years in two patients. When comparing these 20 patients with documented past HEV infection to the 123 without, there was a difference in the frequency of HEV IgG positivity, albeit a statistically non-significant one (*p* = 0.075). In the first group, HEV IgG was measured and was positive in 19 patients (100%), while in the second, HEV IgG was measured in 122 patients and was positive in 102 (83.6%). Overall, HEV IgM might not be an ideal marker for screening patients for an HEV infection.

### 3.5. Sensitivity and Specificity of Commercially Available HEV IgM ELISA Tests Differ between Immunocompetent and Immunosuppressed Individuals

**Figure 2:**
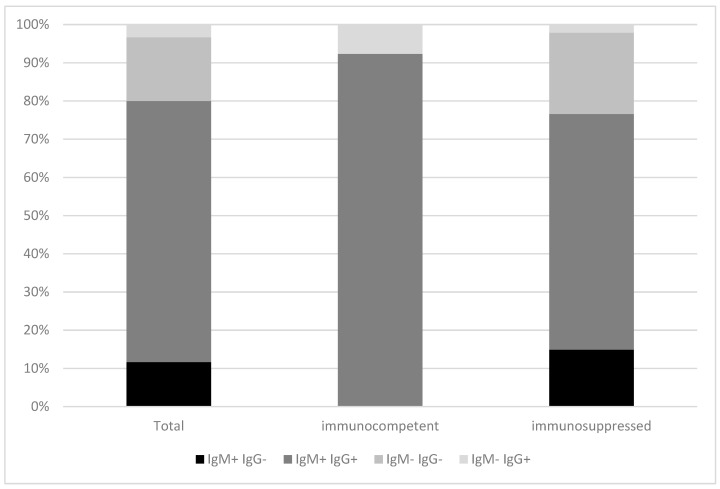
Serological profile of immunocompetent vs. immunosuppressed patients with detectable HEV RNA.

Next, we wanted to evaluate if the lack of detection of HEV-specific antibodies in HEV RNA positive individuals differs between immunocompetent patients and patients under immunosuppression. As shown in Figure 2, HEV IgG antibodies were detectable in all immunocompetent HEV RNA positive individuals (*n* = 13, 100%), while 17 (35.4%) immunosuppressed patients with HEV RNA had no HEV IgG antibodies. HEV IgM was absent in one immunocompetent patient (7.7%) with detectable HEV RNA and in eleven (22.9%) immunosuppressed patients. HEV RNA positive/HEV IgM and IgG negative patients (*n* = 10, 20.8%) were only to be found in the group of immunosuppressed patients (two patients after allogeneic stem cell transplantation, three patients after liver transplantation, one patient after kidney transplantation, one patient with overlap syndrome with primary biliary cholangitis and autoimmune hepatitis under immunosuppressive therapy, two patients with lymphoma and one patient with chronic lymphatic leukemia). Overall, these data indicate that especially in immunosuppressed patients HEV antibody testing without additional detection of HEV PCR is insufficient. In addition, also in immunocompetent HEV RNA positive patients, HEV IgM can be missing.

Overall, when comparing the detection of HEV IgM by Euroimmun ELISA with the “gold standard” of HEV RNA detection, immunosuppression was associated with a reduction of sensitivity and specificity of the HEV IgM ELISA test. The estimated sensitivity, specificity, positive and negative predictive value (PPV and NPV, respectively) for the total of our patient cohort and the immunocompetent and immunosuppressed subgroups are shown in Table 3. Especially the reduced sensitivity in immunocompromised individuals indicated that at least in this patient cohort HEV RNA testing was mandatory to confirm diagnosis of HEV infection.

### 3.6. A Specific HEV IgA ELISA Fails to Detect IgA in HEV RNA Positive/HEV IgM Negative Patients

**Figure 3:**
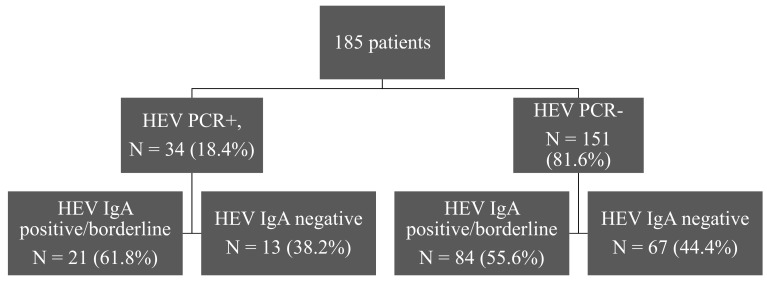
Distribution of HEV PCR positive and negative patients according to their HEV IgA status.

Since HEV is typically transmitted fecal–orally, we wondered if HEV IgA testing would be more sensitive, especially in patients without detectable HEV antibodies or with suspected false positive HEV IgM detection. Therefore, we tested available sera from all four groups of patients for detection of HEV IgA, as shown in Figure 3. HEV IgA antibodies were detectable in about half of the patients without detectable HEV RNA and undetectable in 38% of the patients with detectable HEV RNA. The sensitivity of the method was 61.76 % (95% CI, 43.56–77.83) and the specificity 44.37% (95% CI, 36.3–52.67).

HEV IgA was undetectable in all HEV RNA+/HEV IgM− patients. In HEV RNA−/HEV IgM+ patients, most individuals also had detectable HEV IgA antibodies, as shown in Table 4 in detail. Overall, these data indicate that HEV IgA testing is not a more sensitive marker than HEV IgM for the diagnosis of HEV infection.

## 4. Discussion

Previous studies have evaluated the diagnostic performance of different serological assays for HEV IgG, IgM and to a lesser degree HEV IgA [11,12]. Variability in the performance of such assays is common [13,14,15,16], but false positive cases in healthy blood donors and false negative cases in patients with acute hepatitis seem to be rare [17,18]. In our cohort, diagnosis of HEV infection was sufficient in immunocompetent patients when testing for both HEV IgM and IgG antibodies.

In immunosuppressed patients, however, things looked different; when comparing the diagnostic accuracy of HEV IgM with the “gold standard” HEV RNA, immunosuppression was associated with reduced sensitivity (76.6 vs. 92.9) and specificity (85 vs. 89.4) compared to the immunocompetent cohort. Inferior performance is not surprising, since poor antibody response/formation has been repeatedly described in immunosuppressed patients previously, an effect observed not only after a natural infection but also after vaccination [19,20,21]. Indeed, immunocompetent HEV RNA positive patients had higher serum total IgG concentration and HEV IgM titers than immunosuppressed HEV RNA positive patients, although the difference in HEV IgM titers did not reach statistical significance (*p* = 0.056). Thus, relying only on serology (HEV IgM positivity) in immunosuppressed patients would lead to an unacceptable number of misdiagnosed cases, in terms of both false negative but also false positive results. 

The difference in the severity and fatality of the HEV infection in the above mentioned groups can be attributed to selection bias. Usually, only relatively severe cases of hepatitis are being tested for HEV RNA and/or sent to a tertiary hospital like the University Hospital of Essen, while the threshold of testing for HEV RNA in immunocompromised hosts is lower. 

Stratification and comparison of patients with replicative HEV infection according to their HEV IgM status showed no significant differences between the groups in age, clinical background, ALT levels, HEV viral load or outcome. The only significant difference was observed in the total serum IgG concentration, which was greater in the group with detectable HEV IgM. 

HEV IgA and IgM antibodies appeared at the same time point after infection in serum; the duration of IgA seropositivity, however, slightly exceeded the duration of IgM seropositivity [22]. In order to investigate if the diagnostic accuracy of HEV serology could be improved with the use of HEV IgA testing, we tested available sera from all four groups of patients for HEV IgA. The results were not encouraging for HEV RNA+/HEV IgM− patients; HEV IgA was undetectable in all of them. In the case of HEV RNA−/HEV IgM+ patients, most but not all patients had detectable HEV IgA as well. Thus, no obvious benefit could be observed when performing the HEV IgA assays in combination with HEV IgM and IgG detection in our cohort.

Limitations of our study are its retrospective character and the relative bias due to recruitment of patients from a tertiary hospital. The samples were tested only with the Euroimmune HEV IgM and IgG serological assay (with the exception of the HEV IgA tested samples). This is a limitation of our study, since different assays have demonstrated different and sometimes higher diagnostic accuracy. Amongst them is the Wantai assay, which has been often but not always regarded as the “gold standard” in HEV serology [22]. Furthermore, we did not evaluate the HEV IgG avidity in the HEV RNA−/HEV IgM+ patient group, which might have helped to differentiate between patients with resolved HEV infection and patients with nonspecific antibodies cross-reacting with the HEV antigen [23]. A possible caveat, however, is that HEV antibody maturation in immunosuppressed hosts may not follow the same pattern as in immunocompetent hosts, as it has been shown for CMV antibodies [24]. Generalizing these results and applying them to our cohort with immunosuppressed patients may not be appropriate. A strength of the study is the relatively great number of tested patients and in particular immunosuppressed patients. Diagnosing viral infection or reactivation during immunosuppression is crucial for the optimal treatment of patients, but serological markers can prove sometimes unreliable, as it has been reported for cases with EBV or HBV infection [25,26]. As we have demonstrated, the same is true for HEV infection. Performing a PCR to diagnose or exclude HEV infection is necessary for immunocompromised patients. This is of great importance for the clinical routine; HEV diagnosis or exclusion is purely serology-based in many cases in Germany, a strategy promoted by the lack of financial compensation for HEV PCR.

This is not necessarily true for immunocompetent hosts though. Although older serological assays for HEV diagnosis had come in disrepute due to their unacceptable performance, newer assays perform as shown in our and previous studies reasonably well [2,11,12,22]. According to our data, performing HEV PCR in immunocompetent hosts would be advisable only if the HEV serology (IgM or/and IgG) is positive. According to the current EASL guidelines, the performance of an HEV nucleic acid diagnostics is recommended to diagnose HEV, ideally but not necessarily in combination with HEV serology [2]. This is understandable from a European perspective; detection through PCR is reliable, sensitive, specific and readily available. This, however, may not reflect the global situation. In developing countries, HEV remains a major public health concern, causing large waterborne epidemics that are associated with high morbidity and mortality [27,28,29]. In such countries and situations, resources and availability of specialized laboratory equipment may be limited. A purely serological diagnosis would thus seem an acceptable compromise when dealing with immunocompetent hosts.

In conclusion, the HEV antibody testing by ELISA performed reasonably well compared to HEV RNA detection in immunocompetent hosts, but less so in immunosuppressed patients. The performance of HEV nucleic acid diagnostics is necessary to diagnose or exclude HEV infection in immunocompromised hosts.

## Figures and Tables

**Table 1:** Clinical, laboratory and virological characteristics of immunocompetent and immunosuppressed HEV RNA positive patients.

	Immunocompetent Patients *n* = 13	Immunosuppressed Patients *n* = 48	*p*
Age in years	56.5 (46–61.5)	51 (26–58)	**0.044**
Sex (m/f)	12/1	35/13	0.264
HEV RNA at diagnosis, IU/mL	1.06 × 10^6^ (0.41 × 10^6^–5 × 10^6^)	2.27 × 10^6^ (0.1 × 10^6^–5 × 10^6^)	0.899
ALT, IU/mL *n* = 58	2351 (1546–3443)	138 (38–413)	**<0.001**
ALT > ULN, n,% *n* = 59	13 (100%)	38 (82.6%)	0.180
IgG, g/L *n* = 29	13.1 (9.9–14.6)	8.9 (6.95–12.2)	**0.011**
ANA positivity, n, % *n* = 24	7 (63.6%)	3 (23%)	0.095
Death/Survival	4/9	2/46	**0.016**
Liver failure (yes/no)	6/7	1/47	**<0.001**
Chronification (yes/no) *n* = 45	0/5	13/27	0.301
HEV IgM (S/CO) *n* = 60	6.35 (3.12–7.62)	3.72 (0.84–6.68)	0.056
HEV IgG (S/CO) *n* = 60	5.49 (0.88–6.32)	4.19 (0.51–5.59)	0.355
HEV IgA (S/CO) *n* = 34	4.63 (3.39–4.65)	1.33 (0.32–3.59)	**0.012**

Data are presented as median (IQR) for continuous variables and n, percentage for categorical variables. For comparisons between groups, we used Chi Square, Fisher’s exact test, unpaired t-test or Mann–Whitney U. ALT: alanine aminotransferase, ULN: under the normal limit, ANA: antinuclear antibodies.

**Table 2:** Clinical, laboratory and virological data of patients with HEV infection stratified according to their HEV IgM status.

	Total *n* = 61	IgM+ *n* = 49	IgM− *n* = 12	*p*
Age in years	54 (38.5–59)	54 (39–59)	52.5 (36–58)	0.842
Sex (m/f)	47/14	40/9	7/5	0.124
HEV RNA at diagnosis, IU/mL	1.82 × 10^6^ (0.24 × 10^6^–5 × 10^6^)	1.82 × 10^6^ (0.24 × 10^6^–5 × 10^6^)	3.2 × 10^6^ (0.15*10^6^–5 × 10^6^)	0.970
HEV IgM (S/CO), *n* = 60	4.63 (1.54–6.76)	5.96 (3.01–7.13)	0.03 (0.03–0.12)	**<0.001**
HEV IgG (S/CO), *n* = 60	4.25 (0.57–6.11)	5.32 (2.87–6.35)	0.26 (0.19–0.53)	**<0.001**
HEV IgA (S/CO), *n* = 34	2.58 (0.41–4.56)	3.59 (1.96–4.62)	0.16 (0.11–0.32)	**<0.001**
ALT, IU/mL *n* = 58	152 (82–914)	181.5 (86–1021)	134.5 (22–233)	0.253
ALT > ULN, n,% *n* = 59	51 (86.4%)	44 (89.8%)	7 (70%)	0.125
IgG, g/L *n* = 29	10.4 (7.8–12.9) *n* = 29	12.1 (9.2–13.7) *n* = 21	7.1 (6.6–8.9) *n* = 8	**0.004**
ANA positivity, n, % *n* = 24	10 (41.7%)	9 (56.3%)	1 (12.5%)	0.079
Immunosuppression (y/n)	48/13	37/12	11/1	0.432
Neurological symptoms (y/n)	1/60	1/48	0/12	1
Outcome (survival vs. non survival)	55/6	44/5	11/1	1
Existing liver disease (y/n)	15/46	11/38	4/8	0.467
Presence of cirrhosis (y/n)	5/56	5/44	0/12	0.573
Liver failure (y/n)	7/54	7/42	0/12	0.327
Liver transplanted patient (y/n)	9/52	7/42	2/10	1
Chronification (y/n)	13/32	9/27	4/5	0.411
Ribavirin Therapy (y/n)	14/47	13/36	1/11	0.264
CMV reactivation (y/n)	4/36	3/30	1/6	0.552
EBV reactivation (y/n)	20/18	18/14	2/4	0.395
BKV reactivation (y/n)	3/17	3/16	0/1	1

Data are presented as median (IQR) for continuous variables and n, percentage for categorical variables. For comparisons between groups we used Chi Square, Fisher’s exact test, unpaired t-test or Mann–Whitney U. ALT: alanine aminotransferase, ULN: under the normal limit, ANA: antinuclear antibodies, CMV: cytomegalovirus, EBV: Epstein Barr virus, BKV: human polyomavirus 1.

**Table 3:** Sensitivity, specificity, PPV and NPV of HEV IgM vs. HEV RNA detection in serum of immunocompetent and immunosuppressed patients.

	Total	Immunocompetent Patients	Immunosuppressed Patients
Sensitivity, %	80.3 (67.8–89)	92.9 (64.2–99.6)	76.6 (61.6–87.2)
Specificity, %	87 (84.9–88.9)	89.4 (86.3–91.8)	85 (81.8–87.7)
PPV	0.26 (0.2–0.32)	0.19 (0.11–0.31)	0.29 (0.21–0.38)
NPV	0.99 (0.98–0.99)	1 (0.99–1)	0.98 (0.96–0.99)

The numbers in brackets represent the 95% confidence interval. PPV: positive predictive value, NPV: negative predictive value.

**Table 4:** HEV IgA detection in patients with and without detectable HEV IgM.

HEV RNA+		
	HEV IgA−	HEV IgA+ or borderline
HEV IgM−, *n* = 9	9 (100%)	0 (0%)
HEV IgM+, *n* = 25	4 (16%)	21 (84%)
HEV RNA−		
	HEV IgA−	HEV IgA+ or borderline
HEV IgM+, *n* = 84	17 (20.2%)	67 (79.8%)
HEV IgM−, *n* = 67	50 (74.6%)	17 (25.4%)

## Supplementary Materials

The following are available online at https://www.mdpi.com/2076-0817/9/2/137/s1, Table S1: Data of HEV IgM and IgG negative HEV PCR positive patients, Table S2: Clinical, laboratory and virological data of patients without replicative HEV infection stratified according to their HEV IgM status.
