# Supplementary material for: Comprehensive Evaluation of Hepatitis E Serology and Molecular Testing in a Large Cohort
Source: Pathogens. 2020 Feb 19;9(2):137. doi: 10.3390/pathogens9020137 (PMC7168254; doi:10.3390/pathogens9020137)
Supplement: Supplementary file 1 [file pathogens-09-00137-s001.pdf]

Supplement

Table S1: Data of HEV IgM and IgG negative HEV PCR positive patients.

	Sex	Age (years)	Comorbidities	Serology	Outcome
1	f	15	AML, HSCT	HEV IgG-<br>HEV IgM/IgG- in follow up	Chronic HEV infection, no ribavirin therapy
2	m	58	lymphoma, CTx	HEV IgG-	No follow up
3	f	42	PBC/AIH overlap, Cyclosporin/Budesonid	HEV IgG-<br>HEV IgM/IgG+ in follow up	Resolution, HEV IgM
4	m	7	LTx due to Wilson disease	HEV IgG-<br>HEV IgM/IgG+/- in follow up	Chronic HEV infection, ribavirin therapy, resolution
5	f	45	LTx due to PBC	HEV IgG-<br>HEV IgM/IgG+ in follow up	resolution
6	f	64	Lymphoma, CTx	HEV IgG-<br>HEV IgM/IgG- in follow up	resolution
7	m	51	CLL, CTx, liver infiltration	HEV IgG-<br>HEV IgM/IgG- in follow up	short follow up
8	m	54	LTx due to alcoholic cirrhosis	HEV IgG-<br>HEV IgM/IgG- in follow up	resolution
9	f	34	ALL, HSCT	HEV IgG-<br>HEV IgM/IgG- in follow up	resolution
10	m	57	Kidney transplantation	HEV IgG-<br>HEV IgM+/IgG- in follow up	Chronic HEV infection, no ribavirin therapy, resolution

Samples from all patients except patient 9 were tested at the timepoint of HEV diagnosis for detectable HEV IgA, none was positive. M: male, f: female, AML: acute myeloid leukemia, HSCT: human stem cell transplantation, GvHD: graft vs host disease, CTx: chemotherapy, PBC: primary biliary cholangitis, AIH: autoimmune hepatitis, LTx: liver transplantation, NASH: non-alcoholic steatohepatitis, CLL: chronic lymphatic leukemia, ALL: acute lymphatic leukemia.

Table S2: Clinical, laboratory and virological data of patients without replicative HEV infection stratified according to their HEV IgM status.

	IgM+<br>N= 143	IgM-<br>N= 961	p
Age in years	52(45.5-63)	47(33-62)	**0.008**
Sex (m/f)	72 (50.3%)/ 71(49.7%)	553 (57.5%)/ 408 (42.5%)	0.124
Immunosuppression, (y/n)	88(61.5%)/ 55(38.5%)	498(51.8%)/ 463(48.2%)	**0.031**
HEV IgG presence (n=1094)	121 (85.8%)	238 (24.8%)	**<0.001**
HEV IgA presence (n=151)	67 (79.8%)	17 (25.4%)	**<0.001**
